# Diagnostic Impact of Liver Biopsy Among Antinuclear Antibody Positive Individuals With Mild Liver Enzyme Elevation

**DOI:** 10.1111/apt.70665

**Published:** 2026-04-09

**Authors:** David Mehdi Asgher Niazi, Alphonse Charbel, Jan Pfeiffenberger, Uta Merle, Jessica Seessle, Theresa Wenz, Christa Flechtenmacher, Patrick Michl, Conrad Rauber

**Affiliations:** ^1^ Department of Gastroenterology, Hepatology, Infectious Diseases and Intoxication Heidelberg University Hospital Heidelberg Germany; ^2^ Institute of Pathology Heidelberg University Hospital Heidelberg Germany

**Keywords:** antinuclear antibodies, autoimmune hepatitis, complications, liver biopsy, liver enzyme elevation, mild

## Abstract

**Background and Aims:**

Abnormal liver enzyme elevations with positive antinuclear antibodies (ANA) frequently prompt investigation for autoimmune hepatitis after common causes such as viral hepatitis or drug‐induced liver injury have been excluded. Current guidelines recommend liver biopsy for suspected autoimmune hepatitis, but its utility in patients with only mild transaminase elevation remains uncertain. This study evaluated the diagnostic yield of liver biopsy in such patients, particularly when alanine aminotransferase is below 101 U/L.

**Methods:**

We retrospectively analysed 313 adults who underwent percutaneous liver biopsy at the University Hospital Heidelberg between 2019 and 2024 for unexplained liver enzyme elevation including suspected autoimmune hepatitis. Clinical data were extracted, and histology was classified according to the 2022 International Autoimmune Hepatitis Pathology Group consensus recommendations. Patients with malignancy, post‐transplant biopsy, or incomplete data were excluded.

**Results:**

The median age was 49 years; 55.3% were female. Median alanine aminotransferase and aspartate aminotransferase were 161 U/L and 106 U/L, respectively. Antinuclear antibodies were positive in 61.3% of cases. 22.4% of antinuclear antibody positive and 15.7% of antinuclear antibody negative patients had autoimmune hepatitis compatible histology. Among patients with positive antinuclear antibodies and alanine aminotransferase < 101 U/L, only 9% showed autoimmune hepatitis compatible histology and 3.8% (three patients) required long‐term immunosuppression. Two of these three patients had compensated liver cirrhosis.

**Conclusions:**

Liver biopsy provides limited additional diagnostic value in ANA‐positive, non‐cirrhotic patients with alanine aminotransferase < 101 U/L and no alternative suspected aetiology. In such cases, the procedure can be safely deferred, reserving biopsy for higher enzyme elevations or unclear differential diagnoses.

## Graphical abstract

In ANA‐positive patients with suspected autoimmune hepatitis (AIH), liver biopsy rarely altered therapeutic management when alanine aminotransferase (ALT) was below 101 U/L and cirrhosis was absent. These findings suggest that biopsy may be safely deferred in this low‐risk group.

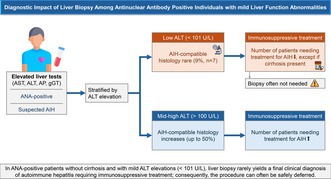

## 1 Introduction

Abnormal liver values are a frequent finding in the general population and represent a common reason for medical consultation [1]. Initial diagnostic work‐up is usually carried out in the general practitioner's setting and includes a thorough medical history as well as basic serological testing to exclude frequent causes of liver enzyme elevation, such as viral hepatitis or excessive alcohol consumption. If these common etiologies are ruled out and elevated liver enzymes prevail, referral to a hepatologist is typically warranted. At the specialist level, further evaluation focuses on excluding rarer liver diseases, including primary biliary cholangitis (PBC), primary sclerosing cholangitis (PSC), and autoimmune hepatitis (AIH) [2]. While some of these conditions can be reliably excluded through blood‐based testing—for example, anti‐mitochondrial antibodies in the case of PBC—the diagnosis of AIH often cannot be established or excluded on serology alone. Early identification of AIH as the underlying cause of elevated liver enzymes is crucial, as it has significant implications for therapeutic management and long‐term prognosis. International guidelines, including the German guideline for rare liver diseases (2024), emphasize the role of histological evaluation of liver tissue in the diagnostic process of AIH, even though this paradigm has been repeatedly challenged [3, 4, 5, 6, 7, 8]. This is reflected in the use of diagnostic scoring systems, such as the simplified autoimmune hepatitis score [9]. However, the threshold of clinical suspicion—particularly regarding the type and degree of liver test (LT) elevation—that should prompt a liver biopsy remains insufficiently defined [10]. Similarly, seronegative AIH constitutes around 10% of AIH patients [11]. As is the case in our tertiary care referral center, positivity of antinuclear antibodies in the context of persistently elevated liver tests commonly prompts percutaneous liver biopsy and histological assessment for autoimmune hepatitis. Since percutaneous liver biopsy is an invasive procedure associated with a small but non‐negligible risk of morbidity and mortality, accurate indication and timing are essential [12, 13]. In this retrospective exploratory cohort analysis, we aimed to better define the diagnostic yield and clinical utility of liver biopsies in patients with unexplained liver enzyme elevations at a tertiary referral center.

## 2 Methods

Using the University Hospital of Heidelberg clinical information system, we identified 1095 liver biopsies performed between 2019 and 2024. The study was approved by the local ethics committee (ethics committee number S‐552/2024). Clinical data including medical history, patient demographics, medications, laboratory values, biopsy technique and reports about complications were subsequently extracted from the clinical information system during the initial screening process.

We aimed to include patients that underwent an ultrasound‐guided percutaneous liver biopsy for the evaluation of elevated liver tests including the routine question to rule out autoimmune hepatitis. Patients undergoing liver biopsy were eligible to be evaluated when any of the liver tests; this included alanine aminotransferase (ALT), aspartate aminotransferase (AST), gamma‐glutamyl transferase (gGT), and alkaline phosphatase (AP) were abnormal. Only liver tests performed within 30 days prior to the biopsy were considered.

**FIGURE 1:**
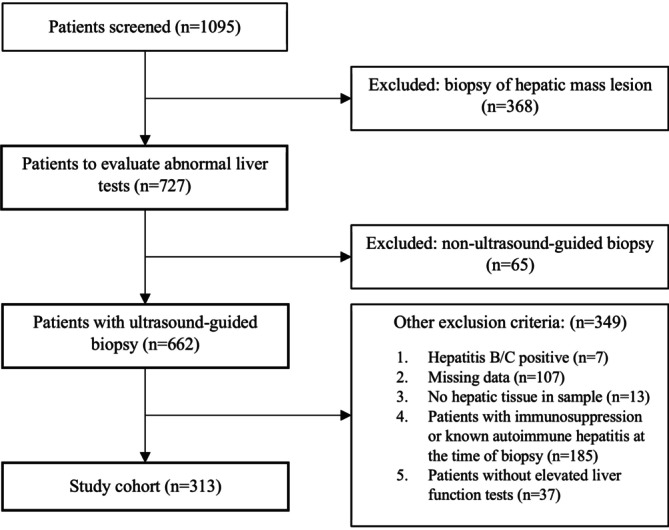
CONSORT diagram of cohort selection. The diagram depicts the selection of patients from all patients undergoing liver biopsy over the study time period.

During the screening process, we excluded all patients who underwent liver biopsy for liver neoplasia, post‐transplant liver biopsy, and patients receiving immunosuppressive therapy at the time of biopsy. In addition, patients with a known diagnosis of AIH were excluded regardless of their current treatment status. Patients with missing data on ALT, AST, or ANA status were also excluded from the study. After applying our inclusion and exclusion criteria, the final cohort comprised 313 patients (Figure 1).

At the University Hospital Heidelberg, liver biopsies were performed by an interventional hepatologist via a percutaneous ultrasound‐guided approach. The formalin‐fixed, paraffin‐embedded tissue was then evaluated by a hepatic pathologist. The final pathological diagnosis was derived from the pathology report. Concerning the histological diagnosis of autoimmune hepatitis, pathology reports were classified according to the consensus recommendations for histological criteria for autoimmune hepatitis from the International Autoimmune Hepatitis Pathology Group (IAIH‐PG) 2022 by an experienced hepatic pathologist. Biopsies prior to the 2022 IAIH‐PG consensus recommendations were reevaluated accordingly. Cirrhosis was defined as the presence of excessive fibrosis, including bridging fibrotic bands and regenerative nodules. Significant fatty liver was defined by the presence of steatosis in more than 5% of hepatocytes. Drug‐induced liver injury (DILI) was pathologically defined as necroinflammatory injury patterns and cholestatic patterns.

### 2.1 Statistical Analysis

IBM SPSS software package version 24.0 was used for data analysis. Baseline characteristics were assessed by median and interquartile range for continuous variables, while totals and frequencies were used for categorical variables, if not indicated otherwise. We used *χ* ^2^ test for qualitative variables, and Fisher exact/Monte Carlo test was used if more than 20% of the cells were below 5 expected count. We used the Student's *t*‐test or the Mann–Whitney *U* test for quantitative variables with a *p* ≤ 0.05 being statistically significant.

## 3 Results

The median (interquartile range) age of 313 patients in the study cohort was 49 (34–58) years, and women represented 55.3% (173) of the study population (Table 1). In 9.9% (31) of patients, cirrhosis was present and diagnosed before the biopsy was performed (Table 1). Median AST was 106 U/L (58–367), ALT 161 U/L (67–549), AP 154 U/L (100–238), gGT 170 U/L (69–341) (Table 1). Median bilirubin levels were 1.3 mg/dL (0.7–6.9) and albumin 41.8 g/L (35.9–45.8). Overall, 61.3% of patients referred to liver biopsy were positive for antinuclear antibodies, 35.1% were strongly positive, defined as ≥ 1:320. IgG values were known in 76.4% of cases, and in 26.8% of cases they were above the upper limit normal (ULN) (Table 1).

**TABLE 1:** Study population.

Variable		Value^a^
Study cohort [*n*]		313
Sex [y]	Male	140 (44.7)
	Female	173 (55.3)
Age [years]		49 (34–58)
Cirrhosis pre‐biopsy		31 (9.9)
Thrombozytes		219 (163–268)
AST [U/L]		106 (58–367)
ALT [U/L]		161 (67–549)
AP [U/L]		154 (100–238)
gGT [U/L]		170 (69–341)
Bilirubin total [mg/dL]		1.30 (0.7–6.9)
Albumin [g/L]		41.8 (35.9–45.8)
International normalized ratio		1.06 (0.99–1.18)
IgG	Unknown	74 (23.6)
	Normal	155 (49.5)
	> ULN	20 (6.4)
	> 1, 1× ULN	64 (20.4)
ANA	Negative	121 (38.7)
	Positive	82 (26.2)
	Strongly positive	110 (35.1)

Abbreviations: ALT, alanine aminotransferase; ANA, antinuclear antibodies; AP, alkaline phosphatase; AST, aspartate aminotransferase; gGT, gamma‐glutamyl transferase; ULN, upper‐limit normal.

^a^ Data are shown as *n* (%) for categorial variables. Continuous variables are presented as median (Q1–Q3).

We stratified patients with LT elevations into ANA‐positive and ANA‐negative patients. Median elevations of LTs were comparable in both groups (Table 2). ANA‐positive and ANA‐negative groups did not differ in positivity for other autoimmune antibodies, including anti‐smooth muscle antibodies (anti‐SMA), antimitochondrial antibodies (AMA), anti‐soluble liver antibodies (anti‐SLA), or anti‐liver‐kidney microsomal antibodies (anti‐LKM) (Table 2). In both cohorts, liver cirrhosis was clinically known in 10.4% and 9.1%, respectively, before biopsy (Table 2).

**TABLE 2:** Clinical characteristics according to ANA positivity in the whole cohort.

Parameter		ANA + (*n* = 192)^a^	ANA − (*n* = 121)^a^	*p* ^b^
ALT [U/L]		155.00 (62.25–510.25)	173.00 (85.00–712.00)	0.117
AST [U/L]		106.50 (56.50–340.25)	106.00 (57.50–430.50)	0.488
gGT [U/L]		169.00 (69.00–334.00)	170.00 (63.00–355.00)	0.835
AP [U/L]		154.00 (98.00–245.00)	154.50 (104.50–234.25)	0.872
IgG	Unknown	47 (24.5)^c^	27 (22.3)	0.367
	Normal	91 (47.4)	64 (52.9)	
	1–1.1× ULN	10 (5.2)	10 (8.3)	
	> 1.1× ULN	44 (22.9)	20 (16.5)	
Anti‐SMA	Positive	15 (7.8)	6 (5.0)	0.283
	Negative	139 (72.4)	83 (68.6)	
	Unknown	38 (19.8)	32 (26.4)	
Anti‐LKM	Positive	1 (0.5)	0 (0.0)	0.750^d^
	Negative	145 (75.5)	88 (72.7)	
	Unknown	46 (24.0)	33 (27.3)	
Anti‐SLA	Positive	5 (2.6)	2 (1.7)	0.709^d^
	Negative	87 (45.3)	60 (49.6)	
	Unknown	100 (52.1)	59 (48.8)	
AMA	Positive	9 (4.7)	11 (9.1)	0.056
	Negative	170 (88.5)	95 (78.5)	
	Unknown	13 (6.8)	15 (12.4)	
Known cirrhosis	Yes	20 (10.4)	11 (9.1)	0.702
	No	172 (89.6)	110 (90.9)	

Abbreviations: ALT, alanine aminotransferase; AMA, antimitochondrial antibodies; anti‐LKM, anti‐liver‐kidney antibodies; anti‐SLA, anti‐soluble liver antibodies; anti‐SMA, anti‐smooth muscle antibodies; AP, alkaline phosphatase; AST, aspartate aminotransferase; gGT, gamma‐glutamyl transferase; ULN, upper‐limit normal.

^a^ Data are shown as *n* (%) for categorial variables. Continuous variables are presented as median (Q1–Q3).

^b^ *p* values calculated using *x* ^2^ test or Mann–Whitney *U* test, as appropriate, unless mentioned otherwise.

^c^ Percentages are calculated within each ANA subgroup (ANA‐positive and ANA‐negative).

^d^ Monte Carlo test was used here as two cells have an expected frequency of less than five.

In Table 3, we have demonstrated clinical characteristics including antibodies relevant to AIH in our subgroup of ANA‐positive patients with only mildly elevated ALT (< 101 U/L). It can be observed that prior to biopsy, ANA‐positive but IgG‐negative patients with ALT < 101 U/L and exclusion of infectious hepatitis comprised 43.6% of the cohort, whereas ANA‐positive and IgG‐positive patients with excluded infectious hepatitis comprised 23.1%.

**TABLE 3:** Clinical characteristics in ANA‐positive patients with ALT < 101 U/L.

Parameter		Value in ANA‐positive patients with ALT < 101 U/L (*n* = 78)^a^
IgG	Unknown	26 (33.3%)
	Normal	34 (43.6%)
	1–1.1× ULN	2 (2.6%)
	> 1.1× ULN	16 (20.5%)
Anti‐SMA	Positive	5 (6.4%)
	Negative	56 (71.8%)
	Unknown	17 (21.8%)
Anti‐LKM	Positive	0 (0.0%)
	Negative	60 (76.9%)
	Unknown	18 (23.1%)
Anti‐SLA	Positive	2 (2.6%)
	Negative	34 (43.6%)
	Unknown	42 (53.9%)
AMA	Positive	2 (2.6%)
	Negative	70 (89.7%)
	Unknown	6 (7.7%)
Known Cirrhosis	Yes	17 (21.8%)
	No	61 (78.2%)

^a^ Data are shown as *n* (%).

### 3.1 Histological Diagnosis

The most frequent histological differential diagnosis in our cohort was drug‐induced liver injury, observed in 51.6% of ANA‐positive and 51.2% of ANA‐negative patients (Table 4). This was followed by steatosis/steatohepatitis (27.6% and 34.7%), autoimmune hepatitis (AIH) possible or likely (22.4% and 15.7%), and cirrhosis (12.5% and 10.7%) in ANA‐positive and ANA‐negative groups, respectively (Table 4). None of these histological patterns differed significantly between groups. However, we did observe a trend between IgG elevation and histological classification. Among the ANA‐positive cohort only 28 (18.8%) of the patients with histological classification “unlikely” had elevated IgG, whereas in the groups “possible” and “likely”, IgG elevation was observed in 21 (55.3%) and 5 (100%) patients, respectively. Common histological differential diagnoses for LT elevations included DILI (51.4%), steatosis or steatohepatitis (30.4%), cirrhosis (11.8%) and chronic cholestatic cholangiopathy (7.7%) (Table 5).

**TABLE 4:** AIH Classification and common histological findings according to ANA positivity.

Histological findings		ANA + (*n* = 192)^a^	ANA − (*n* = 121)^a^	*p* ^b^
AIH	Unlikely	149 (77.6)^c^	102 (84.3)	0.267
	Possible	38 (19.8)	18 (14.9)	
	Likely	5 (2.6)	1 (0.8)	
DILI		99 (51.6)	62 (51.2)	0.956
Steatosis or steatohepatitis		53 (27.6)	42 (34.7)	0.183
Cirrhosis		24 (12.5)	13 (10.7)	0.639
More than mild siderosis		4 (2.1)	5 (4.1)	0.291
Others		27 (14.1)	14 (11.6)	

Abbreviations: AIH, autoimmune hepatitis; ANA, antinuclear antibodies; DILI, drug‐induced liver injury.

^a^ Data are shown as *n* (%) for categorial variables. Continuous variables are presented as median (Q1–Q3).

^b^ *p* values calculated using *x* ^2^ test or Mann–Whitney *U* test, as appropriate, unless mentioned otherwise.

^c^ Percentages are calculated within each ANA subgroup (ANA‐positive and ANA‐negative).

**TABLE 5:** Histological differential diagnoses for liver test elevations.

Histological differential diagnoses	Overall cohort (*n* = 313)^a^, ^b^	ALT < 101 U/L (*n* = 78)^a^, ^b^
DILI	161 (51.4)	23 (29.5)
Steatosis or steatohepatitis	95 (30.4)	28 (35.9)
Cirrhosis	37 (11.8)	21 (26.9)
Chronic cholestatic cholangiopathy^c^	24 (7.7)	7 (9.0)
Infectious causes^c^	12 (3.8)	0 (0.0)
More than mild siderosis	9 (2.9)	2 (2.6)
Secondary cholestatic and ischemic liver injury^c^	9 (2.9)	4 (5.1)
M. Wilson	9 (2.9)	2 (2.6)
Granulomatous hepatitis	8 (2.6)	3 (3.8)
Others^d^	8 (2.6)	4 (5.1)

Abbreviation: DILI, drug‐induced liver injury.

^a^ Data are shown as *n* (%).

^b^ Percentages may exceed 100% and total counts may exceed the number of patients, as up to three histological differential diagnoses could be assigned per patient.

^c^ Category definitions: Chronic cholestatic cholangiopathy included primary sclerosing cholangitis (PSC), primary biliary cholangitis (PBC), and chronic cholangitis of unclear aetiology after exclusion of secondary, infectious, and drug‐induced causes. Infectious causes included viral hepatitis (HAV, HBV, HCV, HEV, EBV) and post‐infectious hepatitis. Secondary cholestatic and ischemic liver injury included secondary sclerosing cholangitis, ICU‐associated cholangiopathy, biliary obstruction or drainage disorders, and ischemic/hypoxic liver injury.

^d^ Others comprised rare causes such as Alpha‐1‐antitrypsin accumulation, aceruloplasminemia, diffuse malignant infiltration, and diffuse nodular hyperplasia.

When ANA‐positive patients were stratified by the magnitude of ALT elevation, the prevalence of AIH (possible or likely) increased with rising ALT levels, from 9.0% in patients with ALT < 101 U/L to 50.0% in those with ALT > 1000 U/L (Table 6). Similarly, the prevalence of DILI as a differential diagnosis rose from 29.5% at ALT < 101 U/L to 88.5% at ALT > 1000 U/L (Table 6). A comparable trend was observed among ANA‐negative patients: the frequency of AIH (possible or likely) increased from 8.1% in ALT < 101 U/L to 33.3% in ALT > 1000 U/L, while DILI rose from 27.0% at ALT < 101 U/L to 81.0% at ALT > 1000 U/L (Table 6). Among patients with ALT > 1000 U/L, hepatitis E infection was detected in four ANA‐positive and two ANA‐negative individuals.

**TABLE 6:** Histological assessment according to consensus recommendations from IAIHG and drug induced liver injury (DILI) as differential diagnosis.

ANA‐status	ALT strata [U/L]		Histological criteria according to IAIH‐PG^a^			DILI as differential diagnosis^a^	
			Unlikely	Possible	Likely	Yes	No
ANA + (*n* = 192)	< 101	78	71 (91.0)^b^	6 (7.7)	1 (1.3)	23 (29.5)	55 (70.5)
	101–200	30	25 (83.3)	4 (13.3)	1 (3.3)	11 (36.7)	19 (63.3)
	201–500	35	25 (71.4)	9 (25.7)	1 (2.9)	23 (65.7)	12 (34.4)
	501–1000	23	15 (65.2)	7 (30.4)	1 (4.3)	19 (82.6)	4 (17.4)
	> 1000	26	13 (50.0)	12 (46.2)	1 (3.8)	23 (88.5)	3 (11.5)
ANA − (*n* = 121)	< 101	37	34 (91.9)	3 (8.1)	0 (0.0)	10 (27.0)	27 (73.0)
	101–200	27	24 (88.9)	3 (11.1)	0 (0.0)	10 (37.0)	17 (63.0)
	201–500	19	18 (94.7)	1 (5.3)	0 (0.0)	11 (57.9)	8 (42.1)
	501–1000	17	12 (70.6)	4 (23.5)	1 (5.9)	14 (82.4)	3 (17.6)
	> 1000	21	14 (66.7)	7 (33.3)	0 (0.0)	17 (81.0)	4 (19.0)

Abbreviations: ALT, alanine aminotransferase; ANA, antinuclear antibodies; DILI, drug‐induced liver injury; IAIH‐PG, International Autoimmune Hepatitis Pathology Group.

^a^ Values are shown as *n* (%).

^b^ Percentages refer to the distribution within each ALT group.

We analysed the suspected causative medications in all patients with DILI as a differential diagnosis, based solely on clinical documentation. A formal causality assessment was not performed. We categorized the medications by drug class in order to facilitate a more concise and interpretable presentation of the results. In more than half of the cases (57.7%), no specific suspected causative agent could be identified due to insufficient documentation, no medication anamnesis or polypharmacy, with more than two potential drugs implicated (Table 7). The two most common implicated drug classes were antibiotics/anti‐infectives and NSAIDs/analgesics, each accounting for 9.3% of DILI cases (Table 7). The single most frequently suspected individual medication was ibuprofen, reported in 11 patients (Table 7).

**TABLE 7:** Suspected causative medications in patients with drug‐induced liver injury as a differential diagnosis (*n* = 161).

Drug class^a^	Medication (*n*)^b^	*n* (%)^c^
Antibiotics/anti‐infectives	Albendazol (2), **Amoxicillin/clavulanic acid (5)**, Brivudine (1), Ceftriaxone (1), Ciprofloxacin (1), Cotrimazol (1), Fosfomycin (1), Isoniazid (2), Penicillin (1)	15 (9.3)
NSAIDs/analgesics	Diclofenac (1), Etoricoxib (1), **Ibuprofen (11)**, Metamizole (2)	15 (9.3)
Cardiovascular drugs and lipid lowering agents	Apixaban (2), **Atorvastatin (3)**, Candesartan/HCT (1), Fenofibrate (1), **Methyldopa (3)**, Ramipril (1), Rivaroxaban (2), Rosuvastatin (1)	14 (8.7)
Antineoplastic/chemotherapeutic agents	Letrozole (1), Lomustin (1), Olaparib (1), Oxaliplatin/capecitabine (1), Paclitaxel (1), **Ribociclib (2)**, Trabectedin (1), Vincristin (1)	9 (5.6)
CNS‐active drugs	Amphetamines (1), Gabapentin (1), Melperon (1), Pregabalin (1), Trimipramin (1), Quetiapin (1), **Sertraline (2)**	8 (5.0)
Immunomodulatory and biologic antineoplastic agents	Infliximab (1), Ipilimumab (1), Nivolumab (1), **Pembrolizumab (2)**, Teriflunomid (1), Trastuzumab (1)	7 (4.3)
Others	Cetirizin (1), Goserelin (1), Herbal and metabolic supplements (2), Metformin (2), Pantozole (1), **Comirnaty COVID‐19 Vaccine (3)**	10 (6.2)
No suspected drug identified/insufficient documentation		53 (32.9)
No single causative drug identified		40 (24.8)

Abbreviations: CNS, central nervous system; HCT, hydrochlorothiazide; NSAIDs, non‐steroidal anti‐inflammatory drugs.

^a^ Within each drug class, the most frequently suspected agent is highlighted in bold.

^b^ Medications were considered suspected based on clinical documentation at the time of evaluation. A formal causality assessment was not systematically performed.

^c^ Percentages refer to the total DILI differential diagnosis cohort and may exceed 100% as patients receiving exactly two potentially causative medications were included as well.

**FIGURE 2:**
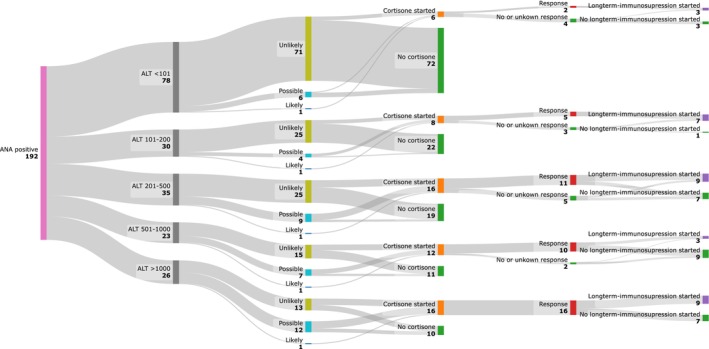
Sankey diagram of ANA‐positive patients stratified by ALT levels and histological AIH classification according to the consensus recommendations from the IAIH‐PG (unlikely, possible, likely) followed by subsequent therapy. Width of the flows correspond to the number of patients. Abbreviations: ANA, antinuclear antibodies, ALT, alanine aminotransferase, IAIH‐PG, International Autoimmune Hepatitis Pathology Group.

In Figure 2 we stratified ANA‐positive patients undergoing liver biopsy according to initial LT elevations and visualized the distribution of AIH evaluation according to IAIH‐PG consensus recommendations and subsequent therapy in a Sankey diagram. In the subgroup of ALT < 101 U/L only 7/78 (9.0%) of patients showed liver histology findings compatible with AIH. In 6 patients (7.7%) probatory corticosteroids were started, resulting in the initiation of three cases of long‐term immunosuppression with azathioprine or mycophenolate mofetil, two of which were patients with compensated liver cirrhosis. In the group with high ALT elevations (> 1000 U/L), corticosteroids were started in 16/26 patients, with a response in all of them, resulting in 9 cases of long‐term immunosuppression consistent with the diagnosis of AIH. In 7 patients, corticosteroids could be tapered subsequently; all of them were finally clinically labelled as DILI.

Biopsy‐related complications included pain in 9 (2.88%) patients, hematoma in 5 (1.60%) patients, of which one required hospitalization and blood transfusion but no intervention to stop bleeding (Table 8). No patient died of liver biopsy in our study cohort.

**TABLE 8:** Liver biopsy complications.

Complication	*n* (%)
Pain	9 (2.88)
Hematoma (Minor)	4 (1.28)
Bleeding with hospitalization and blood transfusion	1 (0.32)
Bleeding with intervention to stop the bleeding	0 (0.00)
Death	0 (0.00)

## 4 Discussion

The objective of this study was to assess the diagnostic utility of liver biopsy in patients presenting with elevated liver tests and positive antinuclear antibodies for the evaluation of autoimmune hepatitis. While current international guidelines recommend liver biopsy when there is clinical suspicion of AIH, the specific parameters defining “clinical suspicion” remain poorly established. Furthermore, AIH can manifest as seronegative disease, lacking ANA positivity or IgG elevation. Consequently, liver biopsy is often performed in patients with elevated liver enzymes and positive ANA titers to confirm or exclude AIH.

Histological evidence supporting a “possible” or “likely” diagnosis of AIH was uncommon among ANA‐positive patients in our cohort, being found in only 43 of 192 individuals (22.4%) (Figure 2). Among those with only mildly elevated ALT (< 101 U/L), AIH‐consistent histology (“possible” or “likely”) was observed in 7 of 78 cases (9.0%), despite high ANA titers in over half of this subgroup (40/78, 51.3%). Only three of these patients with mild ALT elevation were ultimately diagnosed with AIH and started on long‐term immunosuppression. Two of them had cirrhosis and elevated liver stiffness measurements (fibroscan > 20 kPa), consistent with prior studies indicating that advanced fibrosis or cirrhosis in AIH may present with relatively modest enzyme elevations [14]. Detailed serological and histological characteristics of these three patients are provided in Table 9.

**TABLE 9:** ANA, anti‐SMA, IgG and histology in three patients with ALT < 101 treated with long‐term immunosuppression for autoimmune hepatitis.

Patient	ALT (U/L)	ANA	Anti‐SMA	IgG (g/L)	Histological criteria according to IAIH‐PG
1	59	1:160	< 1:20	24.04	Possible
2	78	≥ 1:1280	1:640	44.84	Likely
3	80	≥ 1:1280	1:80	32.54	Unlikely

Abbreviations: ALT, alanine aminotransferase; ANA, antinuclear antibodies; anti‐SMA, anti‐smooth muscle antibodies; IAIH‐PG, International Autoimmune Hepatitis Pathology Group.

As expected, the proportion of AIH‐like histological findings increased with rising transaminases at biopsy. However, even among ANA‐positive patients with ALT > 1000 U/L, cases classified as “AIH likely” remained the minority (3.8%) (Table 6). In contrast, drug‐induced liver injury (DILI) became a more prevalent histological diagnosis with higher ALT levels, aligning with previous evidence that distinguishing AIH from DILI based solely on histology remains challenging [15]. The recognition of this overlap has prompted the concept of drug‐induced autoimmune‐like hepatitis (DI‐ALH), formally described in 2022 [16]. Correspondingly, initiation rates of long‐term immunosuppression as an indication of clinically relevant AIH among ANA‐positive patients rose with increasing LTs, from 23.3% in those with ALT 100–201 U/L to 34.6% in those with ALT > 1000 U/L (Figure 2).

A noteworthy observation in this subgroup with ALT > 1000 U/L was the substantial number of patients with histologically “unlikely” AIH (*n* = 13) who nonetheless underwent probatory corticosteroid therapy (*n* = 7) which led to a biochemical response in all of them (*n* = 7) and to the initiation of long‐term immunosuppression in 28.6% (*n* = 2). This trend mirrors clinical practice patterns, where corticosteroids are empirically employed in cases of persistently elevated LTs of unclear aetiology or in suspected DILI [17].

Our study was not powered to quantitatively evaluate the role of liver biopsy in excluding alternative aetiologies of elevated liver enzymes. However, previous data from comparable cohorts have shown that biopsy yields histopathological findings in up to 85% of cases, though these are often descriptive (e.g., acute or chronic hepatitis) and of uncertain clinical utility to guide treatment [18]. In our series, common alternative histological diagnoses among ANA‐positive patients with mild ALT elevations (< 101 U/L) included steatosis/steatohepatitis (35.9%), cirrhosis (26.9%), chronic cholestatic cholangiopathy (9.0%) and secondary cholestatic and ischaemic liver injury (5.1%) (Table 4).

Percutaneous liver biopsy was overall a safe procedure in this cohort, with complication rates consistent with those reported in the literature and no fatalities reported. Bleeding was the most frequent adverse event, with major haemorrhage observed in just one (0.32%) patient (Table 8).

The main limitations of this study are inherent to its retrospective, single‐centre design, which carries the potential for selection bias and limits generalizability. Additionally, variability in histopathological interpretation may reflect center‐specific diagnostic practices.

In conclusion, histological features consistent with AIH and subsequent clinical diagnoses of AIH were uncommon in non‐cirrhotic patients with mild ALT elevations (< 101 U/L). In this subgroup, liver biopsy should primarily be considered when alternative aetiologies for liver enzyme abnormalities are suspected. Routine biopsy in ANA‐positive, non‐cirrhotic patients with mild LT derangements appears to be of limited diagnostic yield and may not be immediately warranted in the absence of additional clinical indicators of AIH.

## Author Contributions

**David Mehdi Asgher Niazi:** writing – original draft, writing – review and editing, conceptualization, investigation, methodology, visualization, data curation, validation, formal analysis. **Alphonse Charbel:** investigation, validation, writing – review and editing. **Jan Pfeiffenberger:** conceptualization, methodology, writing – review and editing. **Uta Merle:** conceptualization, writing – review and editing. **Jessica Seessle:** conceptualization, writing – review and editing. **Theresa Wenz:** conceptualization, writing – review and editing. **Christa Flechtenmacher:** investigation, validation, resources, writing – review and editing. **Patrick Michl:** conceptualization, methodology, writing – review and editing, supervision, resources. **Conrad Rauber:** writing – original draft, writing – review and editing, conceptualization, investigation, methodology, visualization, data curation, validation, formal analysis, project administration, resources, supervision.

## Funding

The authors have nothing to report.

## Ethics Statement

S‐552/2024, ethics committee Heidelberg University.

## Consent

No patients or the public were involved in the design, conduct, reporting, or dissemination plans of this research.

## Conflicts of Interest

Conrad Rauber—honoraria lectures for BMS, support for attending meetings from AbbVie, receives research grants from the Elke‐Fresenius‐Kroener foundation, German Research Foundation (DFG), and German Cancer Research Center (DKFZ). Patrick Michl—receives consulting fees from AstraZeneca, honoraria lectures for Falk, AstraZeneca, Roche. Theresa Wenz—honoraria lecture for Gilead. All other authors declare no conflicts of interest.

## Data Availability

The data that support the findings of this study are available on request from the corresponding author. The data are not publicly available due to privacy or ethical restrictions.

## References

[1] S. Radcke , J. F. Dillon , and A. L. Murray , “A Systematic Review of the Prevalence of Mildly Abnormal Liver Function Tests and Associated Health Outcomes,” European Journal of Gastroenterology & Hepatology 27, no. 1 (2015): 1–7, 10.1097/MEG.0000000000000233. 25380394

[2] P. Y. Kwo , S. M. Cohen , and J. K. Lim , “ACG Clinical Guideline: Evaluation of Abnormal Liver Chemistries,” American Journal of Gastroenterology 112, no. 1 (2017): 18–35, 10.1038/ajg.2016.517. 27995906

[3] M. Sebode , C. Hudert , A. W. Lohse , and P. Bufler , “Autoimmune and Genetic‐Cholestatic Liver Diseases: The New Guidelines,” Deutsche Medizinische Wochenschrift 1946 150, no. 17 (2025): 1006–1012, 10.1055/a-2462-8656. 40774300

[4] D. Gleeson , R. Bornand , A. Brownlee , et al., “British Society of Gastroenterology Guidelines for Diagnosis and Management of Autoimmune Hepatitis,” Gut 74, no. 9 (2025): 1364–1409, 10.1136/gutjnl-2024-333171. 40169244 PMC12421125

[5] European Association for the Study of the Liver , “EASL Clinical Practice Guidelines on the Management of Autoimmune Hepatitis,” Journal of Hepatology 83, no. 2 (2025): 453–501, 10.1016/j.jhep.2025.03.017. 40348684

[6] E. S. Björnsson and G. M. Hirschfield , “The Role of Liver Biopsy in Diagnosing Patients With Autoimmune Hepatitis ‐ It Can Be Acceptable to Skip!,” Journal of Hepatology 83 (2025): S0168‐8278(25)02315–3, 10.1016/j.jhep.2025.06.017. 40609798

[7] M. Polat , S. Wahlin , and C. Efe , “Can Autoimmune Hepatitis Be Diagnosed and Treated Without Liver Biopsy?,” Journal of Hepatology 84 (2025): e91–e92, 10.1016/j.jhep.2025.09.032. 41077149

[8] E. Bjornsson , J. Talwalkar , S. Treeprasertsuk , M. Neuhauser , and K. Lindor , “Patients With Typical Laboratory Features of Autoimmune Hepatitis Rarely Need a Liver Biopsy for Diagnosis,” Clinical Gastroenterology and Hepatology 9 (2010): 57–63, 10.1016/j.cgh.2010.07.016. 20723617

[9] E. M. Hennes , M. Zeniya , A. J. Czaja , et al., “Simplified Criteria for the Diagnosis of Autoimmune Hepatitis,” Hepatology 48, no. 1 (2008): 169–176, 10.1002/hep.22322. 18537184

[10] M. Sebode , C. Weiler‐Normann , T. Liwinski , and C. Schramm , “Autoantibodies in Autoimmune Liver Disease‐Clinical and Diagnostic Relevance,” Frontiers in Immunology 9 (2018): 609, 10.3389/fimmu.2018.00609. 29636752 PMC5880919

[11] S. A. Bhumi and G. Y. Wu , “Seronegative Autoimmune Hepatitis,” Journal of Clinical and Translational Hepatology 11, no. 2 (2023): 459–465, 10.14218/JCTH.2022.00235. 36643052 PMC9817061

[12] J. Neuberger , J. Patel , H. Caldwell , et al., “Guidelines on the Use of Liver Biopsy in Clinical Practice From the British Society of Gastroenterology, the Royal College of Radiologists and the Royal College of Pathology,” Gut 69, no. 8 (2020): 1382–1403, 10.1136/gutjnl-2020-321299. 32467090 PMC7398479

[13] M. Graf , C. Graf , S. Ziegelmayer , et al., “Complications of Image‐Guided Liver Biopsies: Results of a Nationwide Database Analysis,” PLoS One 20, no. 5 (2025): e0323695, 10.1371/journal.pone.0323695. 40455799 PMC12129161

[14] A. Laschtowitz , K. Zachou , V. Lygoura , et al., “Histological Activity Despite Normal ALT and IgG Serum Levels in Patients With Autoimmune Hepatitis and Cirrhosis,” JHEP Reports 3, no. 4 (2021): 100321, 10.1016/j.jhepr.2021.100321. 34381983 PMC8333110

[15] A. Suzuki , E. M. Brunt , D. E. Kleiner , et al., “The Use of Liver Biopsy Evaluation in Discrimination of Idiopathic Autoimmune Hepatitis Versus Drug‐Induced Liver Injury,” Hepatology 54, no. 3 (2011): 931–939, 10.1002/hep.24481. 21674554 PMC3192933

[16] R. J. Andrade , G. P. Aithal , Y. S. de Boer , et al., “Nomenclature, Diagnosis and Management of Drug‐Induced Autoimmune‐Like Hepatitis (DI‐ALH): An Expert Opinion Meeting Report,” Journal of Hepatology 79, no. 3 (2023): 853–866, 10.1016/j.jhep.2023.04.033. 37164270 PMC10735171

[17] N. P. Chalasani , H. Maddur , M. W. Russo , R. J. Wong , K. R. Reddy , and Practice Parameters Committee of the American College of Gastroenterology , “ACG Clinical Guideline: Diagnosis and Management of Idiosyncratic Drug‐Induced Liver Injury,” American Journal of Gastroenterology 116, no. 5 (2021): 878–898, 10.14309/ajg.0000000000001259. 33929376

[18] A. Khalifa , D. N. Lewin , R. Sasso , and D. C. Rockey , “The Utility of Liver Biopsy in the Evaluation of Liver Disease and Abnormal Liver Function Tests,” American Journal of Clinical Pathology 156, no. 2 (2021): 259–267, 10.1093/ajcp/aqaa225. 33693456 PMC8259499

